# A Two-Stage Fall Recognition Algorithm Based on Human Posture Features

**DOI:** 10.3390/s20236966

**Published:** 2020-12-05

**Authors:** Kun Han, Qiongqian Yang, Zefan Huang

**Affiliations:** School of Traffic & Transportation Engineering, Central South University, Changsha 410000, China; yangqiongqian@csu.edu.cn (Q.Y.); blacktea@csu.edu.cn (Z.H.)

**Keywords:** fall detection, OpenPose, deflection angles, spine ratio, human posture features, classification

## Abstract

Falls are seriously threatening the health of elderly. In order to reduce the potential danger caused by falls, this paper proposes a two-stage fall recognition algorithm based on human posture features. For preprocessing, we construct the new key features: deflection angles and spine ratio to describe the changes of human posture based on the human skeleton extracted by OpenPose. In the first stage, based on the variables: tendency symbol and steady symbol integrated by the scattered key features, we divide the human body state into three states: stable state, fluctuating state, and disordered state. By analyzing whether the body is in a stable state, the ADL (activities of daily living) actions with high stability can be preliminarily excluded. In the second stage: to further identify the confusing ADL actions and the fall actions, we innovatively design a time-continuous recognition algorithm. When human body is constantly in an unstable state, the human posture features: compare value γ, energy value ε, state score τ are proposed to form a feature vector, and support vector machine (SVM), K nearest neighbors (KNN), decision tree (DT), random forest (RF) are utilized for classification. Experiment results demonstrate that SVM with linear kernel function can distinguish falling actions best and our approach achieved a detection accuracy of 97.34%, precision of 98.50%, and the recall, F1 score are 97.33%, 97.91% respectively. Compared with previous state-of-art algorithms, our algorithm can achieve the highest recognition accuracy. It proves that our fall detection method is effective.

## 1. Introduction

Falls are the primary threat to the health and safety of the elderly [1]. According to The WHO Global Report on Falls Prevention in Older Age [2], about 30% of the elderly over 60 years old experience at least one fall accident each year. The “Old People’s Anti-Dumping Joint Tips” issued by The China Association of Gerontology and Geriatrics pointed out that falls are the major cause of death for people over the age of 65, and 50% of the elderly’s injuries are caused by falls. The US Preventive Medicine Working Group (USPSTF) [3] indicated that in 2014, 28.7% of community-dwelling adults 65 years or older had been reported falling, resulting in 29 million falls and an estimated 33,000 deaths in 2015.

The trend of population aging further expands the adverse effects of falls. According to the 2019 revised World Population Prospects: by 2050, the number of persons aged 80 years or over is expected to triple, from 143 million in 2019 to 426 million; the proportion of world’s population over 65 will increase from 9% in 2019 to 16%; one in four persons living in Europe or North America could be aged 65 or over.

Getting timely assistance after a fall can reduce the risk of hospitalization by 26% and death by over 80% [4]. As most elderly fallers cannot return to standing position and seek help immediately on their own, a reliable fall detection method can help the elderly to get timely medical treatment and reduce physical damage. The mainstream fall detection methods can be classified into three categories [5]: wearable sensors-based method, ambient device-based method, and computer vision-based method.

Wearable sensors-based method generally relies on sensor devices, such as gyroscopes and accelerometers, to collect data like acceleration and deflection angles [6,7,8,9,10]. This method has obvious drawbacks for the need to wear out the sensors: wearing the equipment for a long time can be uncomfortable, and seniors with poor memory may forget to wear the equipment. These defects limit the practicality and generalizability of the method.

Ambient device-based method [11,12,13,14] deploys external sensors in the surveillance area to obtain environmental data such as pressure, vibration, and infrared arrays. This type of method is limited to the place where the monitoring equipment is installed. Furthermore, monitoring implementation is hard for the high cost of professional external sensors. Recent years, Wi-Fi devices-based fall detection methods had received widespread attention due to the advantages of low cost and wide availability. However, similar to other ambient device-based methods, susceptibility to environmental interference will affect detection accuracy and robustness.

The computer vision-based methods extract human features from images to recognize falls. Benefiting from the development of computer vision technology, plenty of research [15,16] has been done to develop systems for highly-accurate, user-friendliness automatic fall detection. This type of method also requires the installation of additional equipment, but it is high-precision, easy to use, and minimally restricting user’s activities. With the development of computing power of edge devices, video information analysis can be directly pre-processed locally. After the privacy-related content is removed, the data will be uploaded to the server for further analysis, which can solve the privacy disputes of video data. Therefore, we believe that vision-based methods have significant advantages and will have great feasibility and universality in future promotion.

For the computer vision-based methods, the most critical part is how to design features based on image information. The current methods are quite feasible for distinguishing fall actions from walking, standing, and other high-stability actions, but still challenging for ambiguity actions, like bending and lying down. To solve this problem, under the premise of considering economics and universality, this paper proposes a two-stage fall recognition method based on a single RGB camera. The contributions of this work can be summarized as follows:

- In the preprocessing stage, based on the key-points extracted by OpenPose, we designed new key features named spine deflection angle, thigh deflection angles, calf deflection angles, and spine ratio to characterize human posture.
- In the first stage, we propose a novel human body state division algorithm based on the tendency symbol and steady symbol integrate by these scattered key features. The stable activities of daily living (ADL) movements such as walking and standing can be preliminary identified by judging whether human body is in a stable state.
- In the second stage, to accurately eliminate confusing actions, we innovatively proposed a time-continuous recognition algorithm, and further proposed the compare value, energy value, and state score three-dimensional features to train a classifier to determine whether a fall occurred.

Overall, our tracker achieves remarkable performance on the public dataset. Due to the design of machine learning, our method is real-time and easy to transplant to the hardware platform.

The rest of this paper is arranged as follows. Section 2, we give a brief description of vision-based fall detection method. Section 3, the proposed approach for fall detection and classification are outlined in two stages. Section 4, the performance of the proposed method is assessed. Lastly, Section 5 concludes this work.

## 2. Related Work Based on Computer Vision Systems

The computer vision-based methods can provide rich and intuitive scene information to realize accurate and real-time fall detection. This type of method improves the universality and popularization of human fall detection technology. It can be divided into two types according to images: RGB image and depth image based on Kinect camera.

In the field of computer vision, RGB images [17] are the most widely used, including a wide range of information such as color, texture, and shape. Researchers often use geometric representations and statistical methods to extract human features. Rougier et al. [18] proposed a three-dimensional head positioning fall detection algorithm that represented the head as a three-dimensional ellipsoid. The hierarchical particle filter performed tracking judgment using color histograms and shape information. Feng et al. [19] focused on dynamic features, exploiting ellipse fitting to represent the image of pedestrians. They extracted the direction angle, velocity, integral normalized motion energy map, and blending motion features to discriminate falls. Min et al. [20] used a minimum outer rectangle to represent the shape of the pedestrian, and the change in the aspect ratio of the frame represented the change in the shape ratio of the pedestrian to determine the fall. These rough estimation methods did not need to detect the global posture features, and the calculation was more portable, but it had defects such as poor accuracy, high misjudgment rate, and poor implementation in the realization of human fall detection.

Depth images [21] are widely used in the field of motion recognition and classification for they can reflect spatial information of scenes. Lei Yang et al. [22] proposed a fall detection method based on spatio-temporal context (STC) tracking over three-dimensional (3D) depth images captured by the Kinect sensor. The STC algorithm was used to track the position of head and the distance calculated each frame from head to floor. Xue Bingxia et al. [23] obtained the skeletal points coordinates from depth images, and the curvature scale space feature, the morphological characteristics, the trajectories of skeleton points were extracted to compensate that a single feature cannot fully characterize human motion. A multi-model feature selection and fusion algorithm was proposed for fall detection. Based on the depth information from Kinect sensor, Gasparrini et al. [24] proposed an automatic fall detection method for indoor environment. It performed fall detection by evaluating the Euclidean distance between pixel coordinates of the position. To address the problem that human body extraction based on depth sensor performed very poor when occlusion happened, Zhao et al. [25] proposed a human upper body detection approach to extract human head and upper body center to characterize human activities well. However, the depth camera recognition range is limited, and the application scene is constrained.

The current use of RGB images for human fall recognition is a rough estimation of human pose, which is inferior to the methods of using depth images in the accuracy and precision. However, the depth camera has a distance limitation, and authentic depth information cannot be obtained under strong lighting conditions. In recent years, deep learning methods based on convolutional neural networks had shown great capabilities in the field of computer vision, and great progress had been made in realizing accurate estimation of human posture based on RGB images. Cao Z [26] used CPM as a component, constructed the OpenPose network, which had won the first of human posture estimation competition on the COCO in that year. Therefore, this paper introduces a deep convolutional neural network to achieve accurate estimation of human posture based on RGB images. On this basis, effective and accurate body posture features are obtained for identifying fall behavior. With these features, a reasonable human fall recognition method is proposed to achieve accurate human fall identification work and minimize the false detection and false alarm situation.

## 3. Materials and Methods

In the presented approach, we model fall as a state transition process with time continuity, from a stable state to a disordered state, and this transition process can be characterized by several geometric features. For this trait, we divide the fall detection task into two stages to distinguish the fall action from the highly stable ADL actions, and the confusing ADL behaviors step by step.

For preprocessing, key features: deflection angles (spine deflection angle, thigh deflection angles, and calf deflection angles) and spine ratio are imported to characterize human body posture based on the human skeleton model extracted by OpenPose. Then in the first stage, the key features are integrated into two new variables: tendency symbol and steady symbol. Based on the tendency symbol and steady symbol, human movement can be divided into three states: stable state, fluctuating state, and disordered state to distinguish the fall actions from the highly stable ADL actions.

In the second stage, to further exclude the confusing ADL behaviors, we design an algorithm with time continuity. When the human body continues to be in an unstable state, the human posture features: compare value γ, energy value ε, state score τ are proposed to form a feature vector, and an excellent classifier is trained to complete the fall recognition.

### 3.1. Huamn Key Points & Posture Vectors

**Figure 1:**
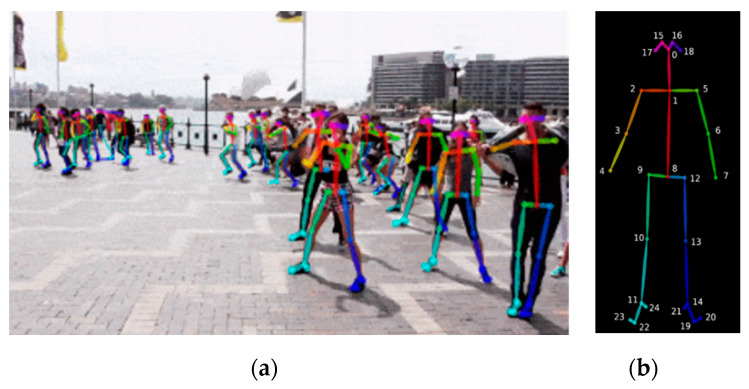
Skeleton extraction based on OpenPose: (**a**) Multi-person skeleton extraction; (**b**) Diagram of the human skeleton key points.

We use OpenPose based on the COCO dataset [27] to extract human body key points, which is the best comprehensive pose extraction method in recent years. OpenPose can obtain 25 key points of human body from 2D image as shown in Figure 1. It not only performs well on RGB images, grayscale images, but also applies to infrared images acquired under weak light conditions. Compared with the methods based on Kinect sensors to obtain key points for fall recognition, the OpenPose-based methods break through the limitation on recognition distance.

**Figure 2:**
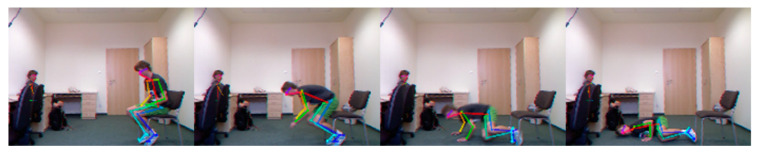
Sequences of falling in the fall-06 video.

**Figure 3:**
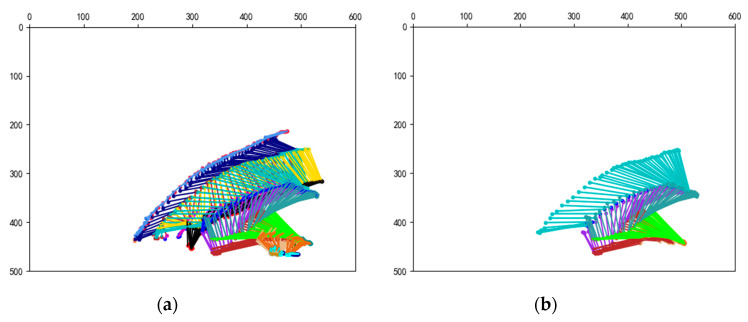
Diagram of human skeleton posture change during the fall processing: (**a**) Complete skeleton extraction; (**b**) Main torso extraction.

Based on OpenPose, we merged the human skeletons of 60 consecutive images corresponding to fall actions (Figure 2) into one image coordinate system. As shown in the Figure 3, removing the arms, wrists, and feet, we can still see an obvious tendency to fall from the main torso composed of the torso, thigh, and calf. In the case of a fall recognition task, the body weight concentrates on the main torso, and the arm, wrist have little influence on its changes. We believe that only focusing on the key points of main torso, an excellent description of human body posture can be obtained, which greatly reduces the calculation of feature extraction.

Based on the human skeleton model obtained by OpenPose, the coordinates of human body’s neck, torso, hip, knee, ankle, etc. in the image coordinate system can be obtained. Then we can calculate the following posture vectors: spine vector (1→8), left thigh vector (9→10), right thigh vector (12→13), left calf vector (10→11), right calf vector (13→14), and waist width vector (9→12). The acquisition method is as follows, and the rest of the vectors can be calculated similarly:

(1) $$\vec{Spine\;\mathrm{vector}}=\left[\begin{array}{c} X_{1}-X_{8}\\ Y_{1}-Y_{8} \end{array}\right]$$

### 3.2. Key Features

As the fall has a consistent and significant tendency, we proposed the deflection angles and spine ratio as key features to describe the change of human posture during the fall behavior and set up experiments to test their performance.

#### 3.2.1. Deflection Angles

We defined the vector parallel to the *y* axis of the image coordinate system as the gravity vector, and the deflection angles are the angles between the posture vectors and the gravity vector, including the spine deflection angle, the thigh deflection angles, and the calf deflection angles. They can directly reflect the deviation of the human torso from the vertical line on the ground [28].

**Figure 4:**
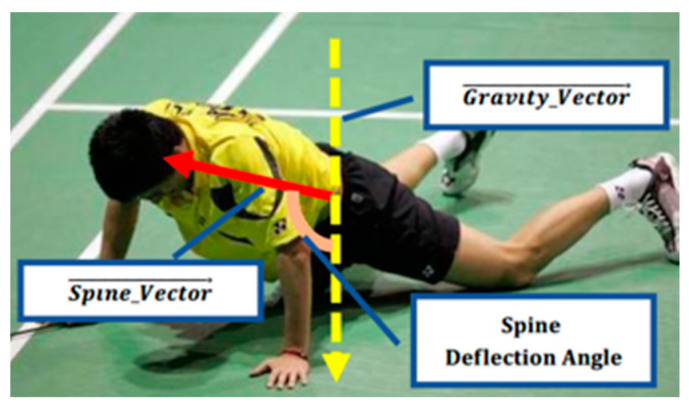
Sketch of the spine deflection angle.

Spine deflection angle is the angle between the spine vector and the gravity vector as presented in Figure 4, which indicates the deviation degree between the human torso and the vertical line of the ground. According to the cosine law, the angle can be calculated by:

(2) $$\begin{array}{c} \cos(spine\;deflection\;angle)=\cos\left(\vec{Spine\;\mathrm{vector}},\;\;\vec{Gravity\;\mathrm{vector}}\;\right)=\\ \;=\frac{\vec{Spine\;\mathrm{vector}}\;*\;\vec{Gravity\;\mathrm{vector}}}{\left|\vec{Gravity\;\mathrm{vector}}\right|\times \left|\vec{Gravity\;\mathrm{vector}}\right|}\; \end{array}$$

Similarly, the (Left, Right) thigh deflection angle and the (Left, Right) calf deflection angle can be obtained, which are used to represent the deviation degree of the thigh and calf relative to the vertical line of the ground.

**Figure 5:**
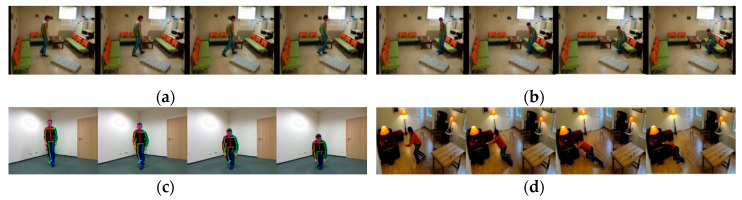
Sequences of four typical actions. (**a**) Walk. (**b**) Sit down. (**c**) Squat. (**d**) Fall.

**Figure 6:**
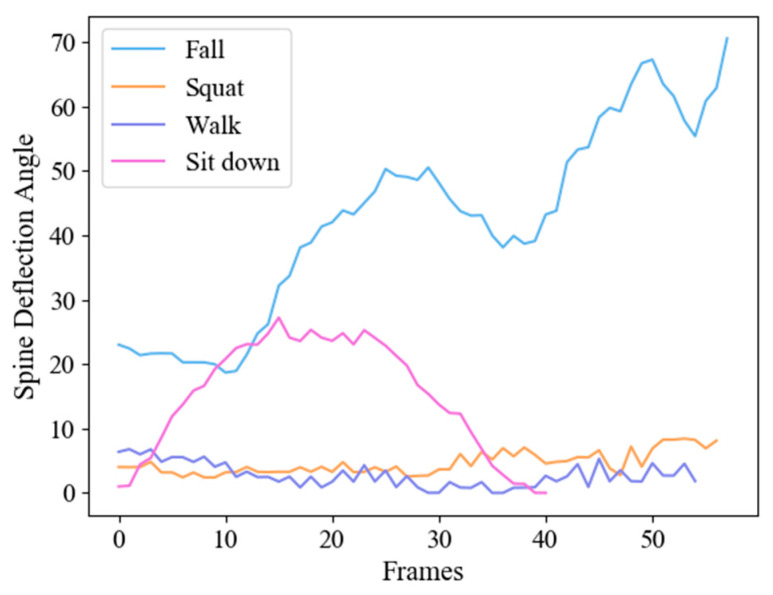
Statistics of spine deflection angle for four typical actions.

**Figure 7:**
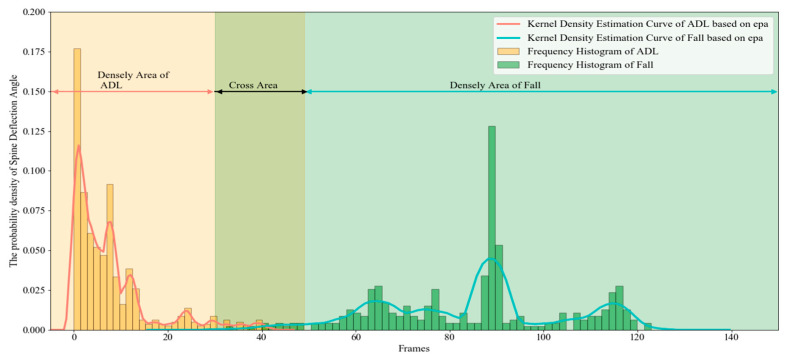
The probability density distribution of the Spine Deflection Angle values in typical movements.

We collect different actions videos taken in multiple scenes (Figure 5) and count the distribution of spine deflection angles in 893 frames. Figure 6 shows the frame-by-frame variation of the spine deflection angles corresponding to typical actions. Figure 7 shows the probability density distribution of the spine deflection angle values in ADL movements and lateral fall movements.

From Figure 6, we can see that when human body in a stable state like walking, the value is small and the fluctuation is gentle; when there is a high possibility of falling, the value is large and fluctuating. The NeuroCom EquiTest [29,30] believes that the stability limit of human body swing tilt angle is 12.5°. It can be observed from Figure 7 that the spine deflection angle values corresponding to the ADL actions is mostly located in the densely area less than 30° and concentrated in the area less than 18°, which meets the NeuroCom EquiTest conclusion. In contrast, the spine deflection angle values of fall actions are mostly distributed in the area greater than 50°. In the Cross Area: 30–50°, there are a few ADL movements and fall movements at the same time. The results mean that the deflection angles can effectively characterize different actions.

#### 3.2.2. Spine Ratio

When human body tends to fall directly in the direction of the camera, it is difficult for the monocular RGB camera to judge the inclination of the human body through the spine deflection angle, we designed the spine ratio as supplement. The spine ratio is the ratio of the spine vector to the waist width vector. It can be calculated by:

(3) $$spine\;ratio=\frac{\left|\vec{Spine\;vector}\right|}{\left|\vec{Waist\;Width\;vector}\right|}$$

**Figure 8:**
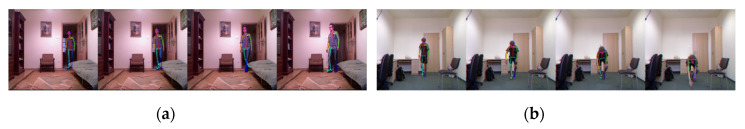
The variation of spine ratio. (**a**) Walk. (**b**) Forward fall.

**Figure 9:**
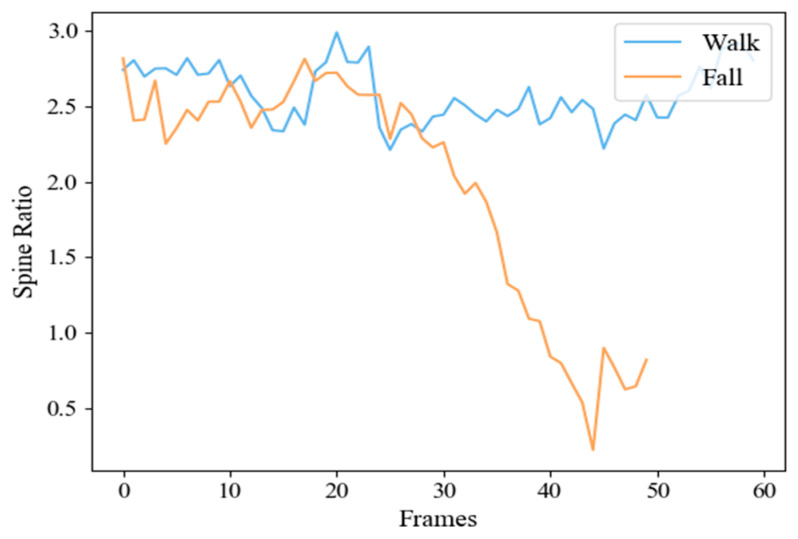
Statistics of spine ratio for walking and a forward fall.

We collected the camera-oriented action sequences (Figure 8) and tested the distribution of spine ratio. The experimental statistics are shown in Figure 9. As shown in the Figure 9, when the human body is in a stable state such as walking, the spine ratio value is large and the fluctuation is gentle; when the human body falls sideways to the camera with his body leaning forward or backward, the value drops rapidly and has severe fluctuations.

**Figure 10:**
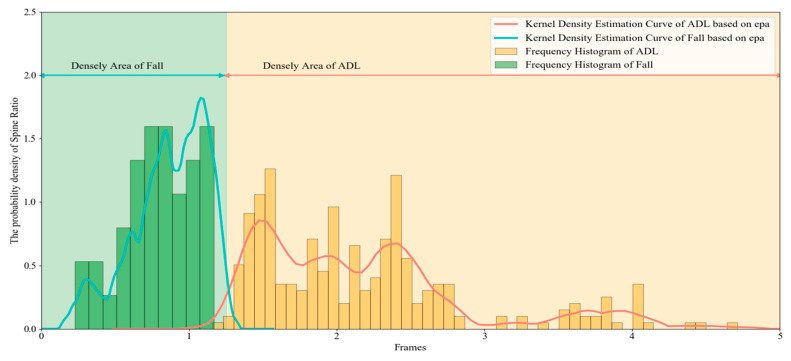
The probability density distribution of the Spine Ratio for walking and a forward fall.

Figure 10 shows the probability density distribution of spine ratio in common ADL movements and forward fall movements. There is a significant difference between these two kinds of actions in the probability density distribution of the spine ratio. The spine ratio corresponding to the fall actions are intensive in the densely area with a value less than 1.2, while the value of ADL actions is intensive in the densely area with a value greater than 1.2. The cross area with ADL behavior and fall behavior is relatively small and negligible.

### 3.3. Primary Fall Recognition: Division of Human Status

The key features are scattered, and a single feature cannot fully characterize the posture of human body. To comprehensively describe the human body posture changes, we integrate multiple key features into the tendency symbol and steady symbol. For fall actions recognition, we classify the human body into three categories: the stable state with a very low possibility of falling, the disordered state with a high possibility of falling and the fluctuating state in between. By distinguishing whether it is in a stable state, stability ADL actions can be recognized initially.

#### 3.3.1. Tendency Symbol

Tendency symbol is used to characterize the body tendency relative to the vertical line of ground. It is integrated from the spine deflection angle and the spine ratio. According to probability density distribution of these two key features, the tendency symbol with three-steps is set. T1 indicates that the human body has a very small inclination angle, such as walking; T2 indicates that the human body has a large inclination angle, such as sits down, leaning over, and the initial stage of falling behavior; T3 indicates that the human body has a significant tendency, and the possibility of falling is extremely high. Algorithm 1 shows the level determination method: ALL (angle lower limit), AUL (angle upper limit) are decided by the probability density threshold of ADL actions and fall actions in Figure 7, and the RB (ratio boundary) depends on the significantly distinguish domains between ADL actions and fall actions in Figure 10.
**Algorithm 1:** the level judgment method of tendency symbol  **Input**: spine deflection angle, spine ratio (ALL = 30°, AUL = 50°; R = 1.0)  **Output**: Tendency Symbol (T1, T2, T3) **If** spine deflection angle <ALL and RB> spine ratio:  Tendency Symbol = T1 **Else If** spine deflection angle >AUL or RB< spine ratio:  Tendency Symbol = T3 **Else**:  Tendency Symbol = T2

#### 3.3.2. Steady Symbol

Steady symbol is used as a supplement to prove whether the body is stable. It is integrated from the deflection angles. When the human body is in a strong stable state, the steady symbol is marked as S1, and when a fall may occur, it is marked as S2. Algorithm 2 shows the level judgment criteria: the AL (angle limit) depends on the probability density distribution of ADL behaviors in Figure 7.
**Algorithm 2:** the level judgment method of stedy symbol  **Input**: spine deflection angle, thigh deflection angles, calf deflection angles, AL = 30°  **Output**: Steady Symbol Case1: spine Angle < AL && left Thigh deflection angle < AL Case2: spine Angle < AL && right Thigh deflection angle < AL Case3: left Calf deflection angle < AL && left Thigh deflection angle < AL Case4: right Calf deflection angle < AL && right Thigh deflection angle < AL **If** Case1 or Case2 or Case3 or Case4:  Steady Symbol = S1 **Else**:  Steady Symbol = S2

**Figure 11:**
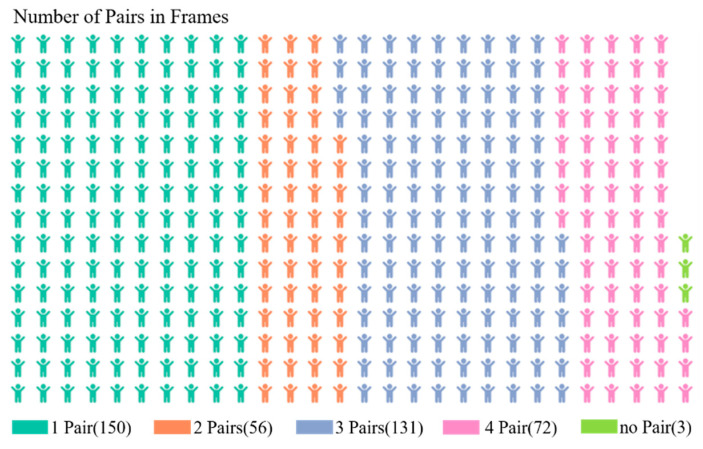
Number of pairs in frames corresponding to activities of daily living (ADL) actions.

We use Pair to describe the case in Algorithm 2, if one of the case1, case2, case3, or case4 is true, we can say that there is a Pair. Based on the collected human action videos, we counted the number of Pairs in 402 frames of ADL movements (upright, walking, squatting, etc.) with strong stability. From Figure 11, it can be observed that the proportion of no-Pair is only 0.73%, and there is at least one Pair in most frames, which means that the corresponding steady symbol will be marked as S1, and human body is stable. The experiment result proves that steady symbol can effectively judge whether the human body is in a relatively stable state.

#### 3.3.3. Division of Human Status

Tendency symbol and steady symbol are the effective integration of scattered independent key features. Based on the symbols, the current state of the human body in the image can be divided into three categories: stable state, fluctuating state, and disorder state, the calculation is shown in Algorithm 3:
**Algorithm 3: division algorithm of human state**  **Input**: Tendency Symbol, Steady Symbol  **Output**: Human State **If** Steady Symbol = S1 && Tendency Symbol = T1:  Human State = stable state **Else If** Steady Symbol = S2 && Tendency Symbol = T3:  Human State = disorder state **Else:**  Human State = fluctuating state

Input the tendency symbol, steady symbol extracted from images into Algorithm 3, If the output is stable state, it can be judged that there is a highly stable ADL action and no fall occurred. Otherwise, we need to perform the second stage of recognition to distinguish the confusing ADL actions and fall actions.

### 3.4. The Second Stage: Recognition of Falling Actions Based on Continuous Human Stage

The human body postures of confusing ADL actions are similar to the pre-fall movements. Judging only by human state based on single image would cause misjudgment. We believe that the unstable state corresponding to fall actions will continue for a certain period time, and we defined it as Non-stationary residence period (NSPR). During the NSRP, there are obvious differences between falls and ADL actions in terms of centroid movement, lower limb stability, and continuous human body status. Therefore, to achieve more accurate fall recognition, we designed a time-continuous fall recognition method, and further proposed the compare-value, energy-value, and state-score to train a classifier to determine whether a fall occurred.

#### 3.4.1. The Update Reference Template Vector and Compare Value

We proposed the vector $\xi =\left[\begin{array}{c} P_{x}\\ P_{y}\\ H \end{array}\right]$ as a reference template. Where $P_{x}$ and $P_{y}$ are the horizontal and vertical coordinate values of key points 8 in the Figure 1, which are close to pelvis region [31]. *H* is the length of the human torso in the image frame. The *H* is calculated as follows:

(4) $$H=\sqrt{{\left\|\vec{Po{\mathrm{int}}_{1}-Po{\mathrm{int}}_{8}}\right\|}_{2}}$$

where $Po{\mathrm{int}}_{1}$ and $Po{\mathrm{int}}_{8}$ indicate the coordinates of the key points 1, 8 in Figure 1.

The compare value γ is used to depict the cumulative change of the human center of mass during the NSPR. When the human body is in steady state, it is marked as 0. It is calculated based on the positions of human centroid and the reference template vector ξ:

(5) $$\mu =softsign\left(P_{y}-Po{\mathrm{int}}_{i_{8y}}\right)*{\left\|\vec{P-Po{\mathrm{int}}_{8}}\right\|}_{2}*H^{-1}$$

(6) $$\gamma =\sum^{\;}\mu$$

where $Po{\mathrm{int}}_{i_{8}}$ refers to the coordinates of the key point 8, and *P* is the position of the stable centroid ($\left[\begin{array}{c} P_{x}\\ P_{y} \end{array}\right]$) in ξ. The softsign function shows vertical movement direction of human centroid during the NSRP.

**Figure 12:**
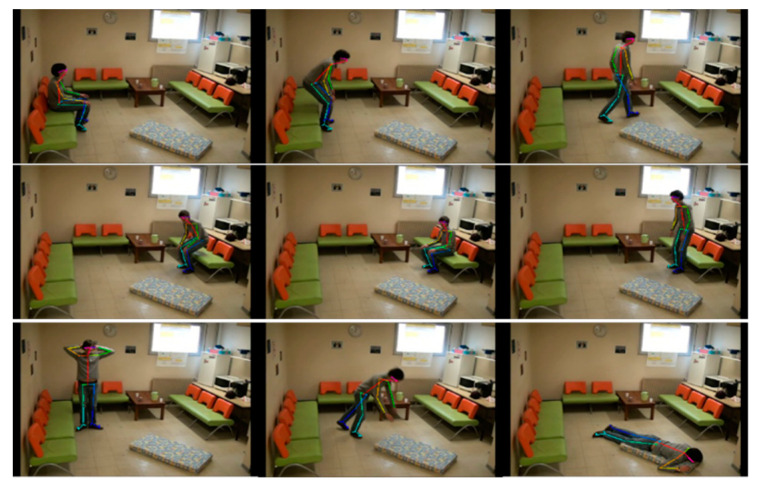
Video sequences from sitting on the sofa-standing-walking-sitting-falling.

**Figure 13:**
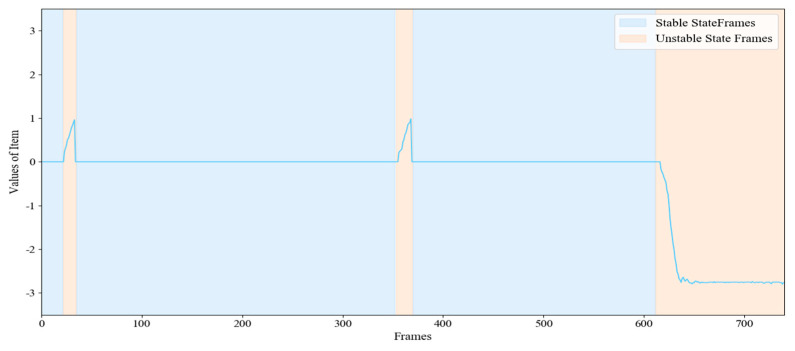
The change of μ corresponding to Figure 12.

Figure 12 shows the frame-by-frame changes of typical human daily activities including ADL movements like sitting on the sofa-standing-walking-sitting and falling movements. Figure 13 shows the change of μ corresponding to Figure 12. In the first 600 frames, most of the value is 0 corresponding to the steady state, and two fluctuations correspond to confusing actions. After the 600 frames, a fall occurs, the value rapidly decreases in the negative direction. As a result, the γ corresponding to ADL actions is significantly different from the fall actions.

#### 3.4.2. Energy Value and State Score

Fall actions are often accompanied by large knee and lumbosacral motions, and the position of the lower limbs will change significantly during the NSRP, which is different from the ADL movements. In order to further eliminate the interference of ADL behaviors, the energy value ε is calculated based on the lower limbs’ energy function to present the stability of the lower limbs as follows:

(7) $$\varepsilon =\sum^{\;}{\left\|\vec{Point_{it_{y}}-\bar{Point_{t_{y}}}}\right\|}_{2}*H^{-1}$$

where $\;\mathrm{Po}int_{it}$ is the vertical coordinate of the key point *t* at the i-th frame, and $\bar{{\mathrm{Point}}_{t_{y}}}$ is the ordinate mean value of key point t during the NSRP. *t* contains the key points 10, 11, 13, and 14. A larger ε represents a higher energy value of the lower limbs, that is, the lower extremities of the human body are more active and less stable, and the probability of a fall is greater.

State score τ refers to the cumulative value corresponding to the human state during the NSRP, as shown in the following formula. The steady state, fluctuating state, and disorder state are assigned as 0, 1, and 2, respectively. τ preserves the distribution of human states during the NSRP. The larger τ, the greater possibility of falling.

(8) $$\tau =\sum^{\;}Human\;State\;Score_{i}$$

**Figure 14:**
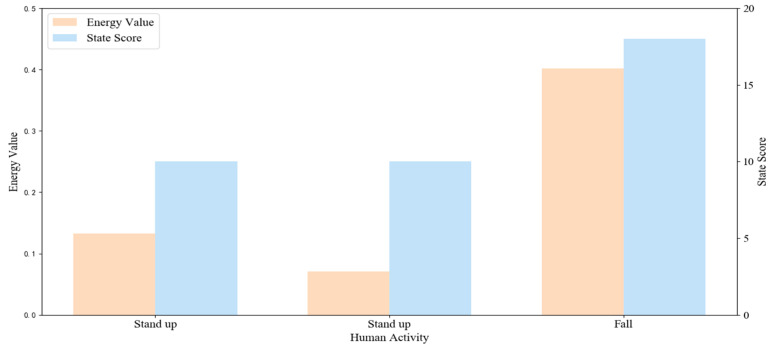
The change of ε and τ corresponding to Figure 12.

Figure 14 shows the typical changes of ε and τ corresponding to Figure 12. We can find that the values of ε and τ are relatively smaller for standing actions, but larger for fall actions. There is a big difference between falling actions and ADL actions, which means that the ε and τ can be used for fall detection.

#### 3.4.3. Recognition Algorithm Based on Continuous Human Stage

On the basis of obtaining human state through the first-stage detection, we designed a time-continuous algorithm shown in Algorithm 4 based on the variables: ξ, γ, ε, and τ to identify fall actions and confusing ADL behaviors.

After obtaining the state of human body, we perform statistics on the state every frame. If there is an unsteady state (fluctuating state, disorder state) in three consecutive frames, we assume that the human body enters NSPR. During NSPR, we record the human body state within 10 frames, similarly, if there is a stable state for three consecutive frames, it is considered that no fall action occurred, we can jump out of NSPR, and restart the statistics. Otherwise, we will form the feature vector $\vec{D}$ by the variables: γ, ε, and τ in 10 frames and input it into the classifier to determine whether the fall behavior occurs. In addition, the reference template ξ needs to be updated in stable state to record the current human posture.
**Algorithm 4:** time-continuous fall recognition algorithm**InPut**: HumanStateFrames[i] **OutPut**: bool IsFall **While** UnstableCount < 3: **If** HumanStateFrames[i] = Stable State:  Update Reference Template Vector ξ;  UnstableCount = 0;  IsFall = False;  Next i; **Else**:  UnstableCount = UnstableCount+1;   UnstableStateFrames.append(HumanStateFrames[i])  Next i; **For** t in [i, i + 10]: **If** HumanStateFrames[i: i + 3] == Stable State  Update Reference Template Vector ξ;  IsFall = False; IsFall = SVM($\vec{D}$), $\vec{D}=\left[\begin{array}{c} \gamma \\ \varepsilon \\ \tau \end{array}\right]$

#### 3.4.4. Classification Based on Machine Learning Methods

We construct a feature vector $\vec{D}$ based on γ, ε and τ during NSRP, and use machine learning methods to implement fall recognition to distinguish the confusing ADL actions from the fall actions. We hope to break the cameras limitations of fall detection. However, the training samples are insufficient for RGB cameras and the real-time requirements are high for the fall recognition task. Compared with the deep learning method, machine learning methods are more suitable and portable for hardware devices. In this paper, four typical classification algorithms: support vector machine (SVM), decision tree (DT), K-nearest neighbors (KNN), and random forest (RF) will be compared and tested, and the optimal method will be selected to realize the fall recognition.

SVM is a supervised machine learning model based on statistical learning theory and structural risk minimization principle. It uses internal kernel functions to project input feature vectors in high-dimensional space, make the features relatively linear, and classify them. It can help to achieve effective classification and determine the best hyperplane in the transformed space to distinguish the input observations. SVM was originally proposed for binary classification and has advantages in small sample pattern recognition [32,33].

SVM has a variety of commonly used kernel functions like linear, poly (polynomial) and RBF (radial basis function).

Linear:

k(x,z)=x∗z

Poly:

$$k\left(x,z\right)={\left(px*z+r\right)}^{u}$$

RBF:

$$k\left(x,z\right)=exp{\left(-p\|x-z\|\right)}^{2}$$

The DT algorithm is a tree structure, where each non-leaf node represents a test on a feature attribute, each branch represents the output of this feature attribute in a certain value range, and each leaf node stores a category. The classification process in decision tree is equivalent to the traversal from the root node to a certain leaf node in the tree. How to traverse each step is determined by the specific attributes of each feature of the data. This article will select the most classic C4.5 [34] algorithm for fall classification and recognition.

KNN is one of the simplest algorithms in data mining classification technology. The core idea of KNN algorithm is that if most of the K nearest neighbor samples of a sample belong to a certain category, the sample also belongs to this category. It achieves classification by measuring the distance between different feature values.

RF is an algorithm based on the DT, which realizes classification by assembling multiple trees. For an input sample, N trees will have N classification results. The RF ensembles all the classification voting results and specifies the category with the most votes as the final output.

## 4. Experiments and Discussion

### 4.1. Dataset

FDD (fall detection dataset) [35] is a public dataset for vision-based fall recognition. It contains 191 videos with a rate of 25 FPS and a resolution of 320 × 240 pixels. The 191 videos of different lengths include various fall actions and ADL actions of different people and multiple camera perspectives in multiple scenes, such as walking, squatting, leaning down, siting and standing, lying down, and falling on the side.

### 4.2. Experimenst on the Frist Stage Algorithm

**Figure 15:**
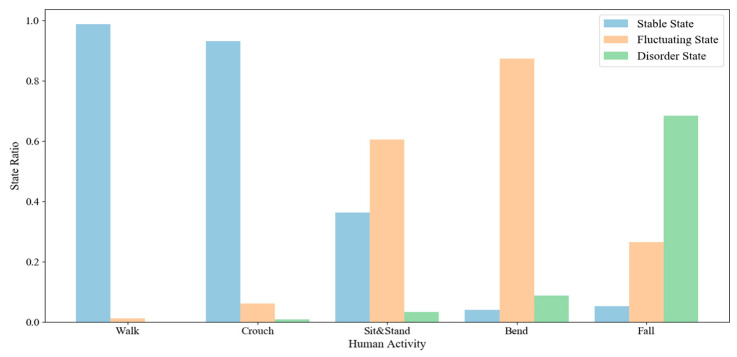
Statistics of human status in various actions.

To verify the effectiveness of the first stage algorithm, we collected multiple scenes, different individuals, multiple types of ADL action and fall action sequences, and statisticsed the distribution of human states. ADL actions were classified in categories such as walk, crouch, sit and stand, bend, etc. The statistical results are shown in Table 1 and Figure 15.

From the statistical results, we can see that the human states corresponding to actions such as walk and crouch were intensive in stable state, and only a small part of them located in fluctuating or disorder state. While the human states corresponding to the falls were denser in the disorder state, and part of it lied in the fluctuating state, it is quite different from the distribution of stable ADL actions. The results show that the first-stage algorithm can distinguish highly stable actions from falling actions well.

In contrast, most of the human states corresponding to actions such as sit and stand were concentrated in fluctuating state. It was difficult to distinguish them from falling actions only by dividing the human state. This kind of confusing ADL action is very similar to the early posture of the fall action, and the second stage of judgment is required.

### 4.3. Experiments on the Second Stage Algorithm

#### 4.3.1. Analysis of the Feature Vector

The classifier in the second stage algorithm uses the γ, ε, and τ during the NSRP as the input feature vector, which is mainly designed for distinguishing falls and highly confusing ADL movements in unstable state. Therefore, ADL movements with high stability such as walking are not used as training samples.

**Figure 16:**
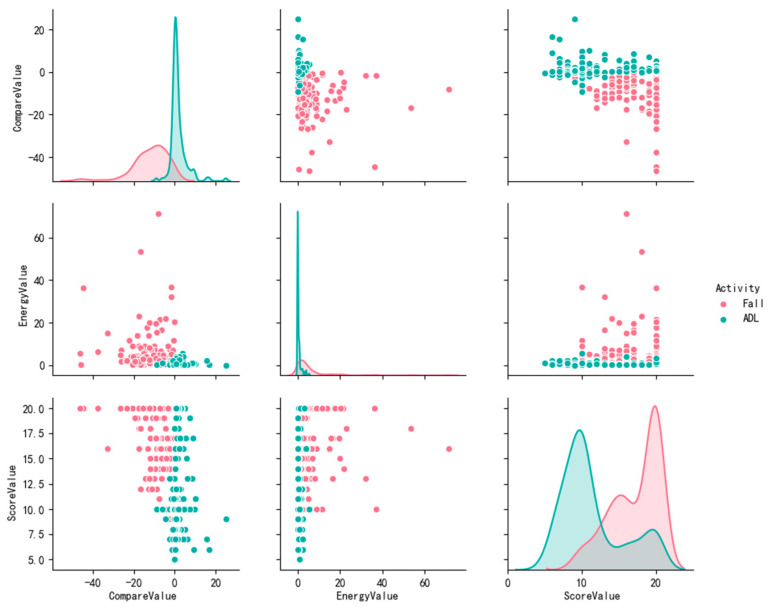
The distribution of the training samples’ feature vectors.

We selected 302 action fragments including 154 fall actions and 148 ADL actions from the FDD dataset. Figure 16 shows the distribution of the training samples’ feature vectors, and the correlation of the variables in the feature vector is represented by a set of two-dimensional matrices.

It can be observed from the Figure 16 that the energy values ε and score scores τ are generally smaller for ADL movements and larger for fall movements. In addition, when falling, the comparison value τ tends to be a large negative value, and the sample point has a higher degree of divergence relative to the ADL action. In general, the attribute overlap of the feature vector is not significant, which is suitable for classification task.

#### 4.3.2. The Classification Results of The Second Stage

**Figure 17:**
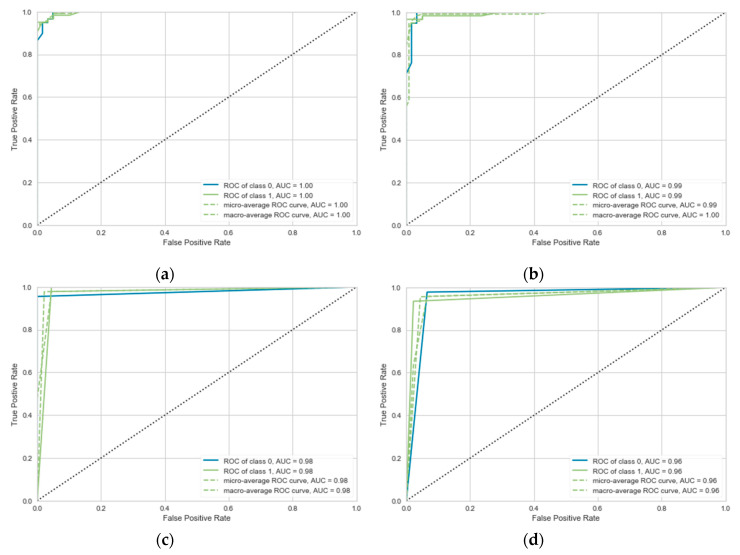
The representation of receiver operating characteristic (ROC) curve. (**a**) Support vector machine (SVM)(Linear); (**b**) random forest (RF); (**c**) SVM(Poly); (**d**) K-nearest neighbors (KNN).

We use Sklearn package to compare the classification effects of seven classifiers: SVM (linear, RBF, poly kernal), KNN, RF, and DT. Where the *p* values of the RBF and poly kernels are set to 1/features (1/3), and the γ value and α value are set to 0, 3. In order to obtain fairer classification evaluation results, we carried out K-fold cross validation. The test results are shown in Figure 17 and Table 2. The evaluation index is the average value of threefold cross validation of each kernel function.

As shown in the Table 2, the performance of SVM with linear kernel is superior to other classifiers, it obtained the highest accuracy, recall, and F1 (0.9668, 0.9622, and 0.9676). The RF achieved the highest precision (0.9671), but it is not outstanding on other evaluation indicators. Figure 17 shows the ROC (receiver operating characteristic) curve of top four classifiers, which can reflect the sensitivity and specificity. From this figure, we can see that the SVM classifier based on the linear kernel achieves best results, all the value of AUC (area under curve) is 1.0. In addition, considering that the non-linear function will take up more computing resources and run time when performing classification prediction tasks, a linear kernel-based SVM classifier is a better choice to compete the classification of falls and confusing ADL action during NSRP.

**Figure 18:**
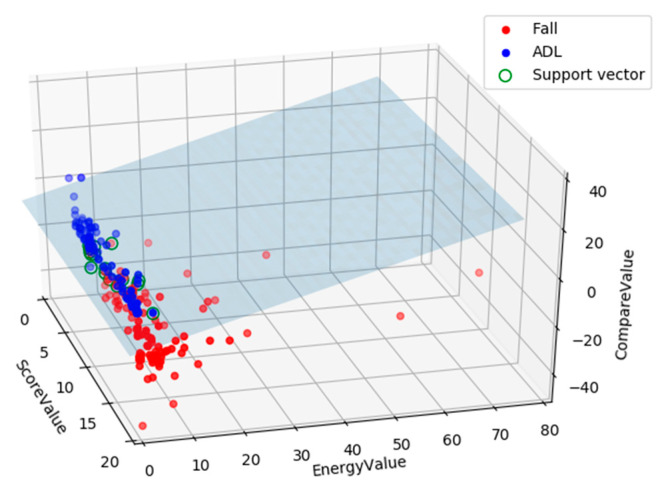
Classification performance of the SVM based on the linear kernel function.

Figure 18 shows the visual classification effect of the SVM based on the linear kernel. We can find that the classification plane has well divided the feature vector points corresponding to the fall actions and the confusing ADL actions. It can be considered that the SVM can perform the classification work excellently, and the fall motion recognition method based on the continuous human state is effective.

### 4.4. Test Results of The Overall Human Fall Recognition Method

The above experiments respectively verified the performance of the first and second stages algorithm. In order to test the performance of the overall algorithm, we conducted experiments on the complete FDD dataset. Table 3 shows the number of different actions identified as non-falling and falling. As can be seen from Table 3, the fall recognition algorithm studied in this paper can well distinguish the fall movement from stable daily activities such as walking and squatting, as well as highly confusing actions such as standing up and bending over. It explains that the proposed method has high fall recognition accuracy and low false detection rate.

**Figure 19:**
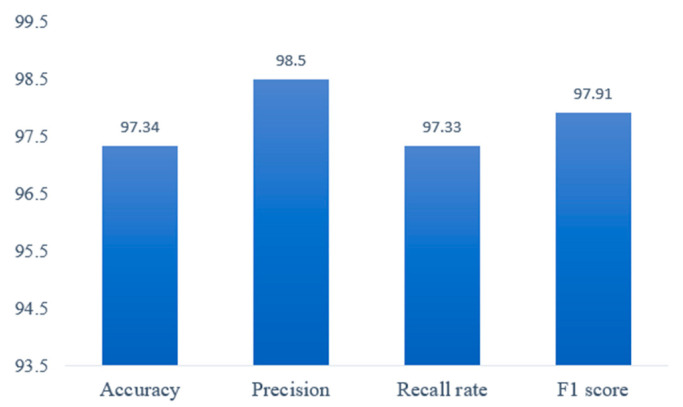
The overall performance evaluation results on Fall Dataset.

Based on Table 3, the overall performance of the algorithm is evaluated in this paper. As shown in Figure 19, he fall recognition method achieves 97.34% accuracy and 98.50% precision, indicating that the method described in this paper can effectively identify the human body’s fall behavior, and the rate of missed detection is low; At the same time, it guarantees a recall rate of 97.33% and a F1 score of 97.91%, indicating that this method has a good distinction between fall behavior and daily activity behavior, and fewer false detections.

Table 4 shows the comparison result with previous state-of-art works [36,37,38] on the same dataset. Since most of the existing fall recognition algorithms only use the accuracy as evaluation indicator, we only conducted a comparative analysis of accuracy. It can be seen from the Table 4 that our method had achieved a higher accuracy rate than previous algorithms. Experiments show that our algorithm has practical value for fall recognition.

## 5. Conclusions

In this paper, we have developed a two-stage fall recognition algorithm based on human posture features. This method based on RGB images can be divided into two parts. In the first part, the highly stable ADL actions is excluded by judging whether the human body is in a stable state with proposed key features; In the second part, when human body is constantly in a fluctuating state or disordered state, the human posture features: compare value, energy value, and state score are introduced characterize the human body’s posture, and SVM is used to realize the classification of confusing ADL actions and fall actions to achieve fall recognition. We have carried out sufficient experiments to verify the effectiveness of the proposed features and algorithms. The performance of the designed mechanism was tested on the publicly available dataset. The results demonstrated that our algorithm not only able to distinguish falls from stable ADL actions but also can separate fall actions and confusing movements like lying and bending over. Compared with other state-of-art fall detection methods, the proposed method has advantage of accuracy.

Our approach mainly considers daily actions in the home environment, and there will still be false detections when dealing with workout motions, like push-ups and burpees. For future work, we will collect more video data to cover as many human actions as possible for algorithm designing and training. Deep learning methods will be incorporated to further improve the recognition accuracy.

## Figures and Tables

**Table 1:** Statistics of human status in various actions.

Actions	Walk	Croch	Sit & Stand	Bend	FALL
Stable State	317	248	142	7	2
Fluctuating State	4	16	237	151	194
Disorder State	0	2	13	15	426

**Table 2:** The preference of several classifiers using the fall detection dataset (FDD) dataset.

Kernel	Accuracy	Precision	Recall	F1 Score
SVM(Linear)	0.9668	0.9636	0.9622	0.9676
RF	0.9381	0.9671	0.8981	0.9339
SVM(Poly)	0.9415	0.9503	0.9112	0.9301
KNN	0.9236	0.9513	0.8886	0.9207
SVM(RBF)	0.8915	0.8556	0.9211	0.9011
DT	0.8805	0.8957	0.8796	0.8715

**Table 3:** Fall recognition test results of the fall dataset.

Actions	Walking	Squat	Stand Up & Sit Down	Bend Over	Falling
Non-Falling	92	23	76	65	4
Falling	0	0	3	4	147

**Table 4:** Performance comparison of several algorithms on the fall dataset.

	Approach	Accuracy
L. Alhimale et al. [36]	Bounding box feature	94.27%
Fouzi Harrou et al. [37]	Fall index area ratios	96.84%
X. Ma e al. [38]	Silhouette, lighting and flow feature	92.17%
Ours	Human posture features Two-stage	97.34%
